# Trends in seroprevalence of influenza A virus infections in pigs in France (2008–2022)

**DOI:** 10.1186/s40813-025-00455-4

**Published:** 2025-07-28

**Authors:** Séverine Hervé, Nicolas Rose, Nicolas Barbier, Stéphane Quéguiner, Stéphane Gorin, Roselyne Fonseca, Gaëtan Pinsard, Gautier Richard, Agnès Jardin, Gaëlle Simon

**Affiliations:** 1 French Agency for Food, Environmental and Occupational Health and Safety (ANSES), Ploufragan-Plouzané-Niort Laboratory, Swine Virology Immunology Unit, National Reference Laboratory for Swine Influenza, Ploufragan, 22440 France; 2 French Agency for Food, Environmental and Occupational Health and Safety (ANSES), Ploufragan-Plouzané-Niort Laboratory, Epidemiology Animal Health and Welfare Unit, Ploufragan, 22440 France; 3 CEVA Santé Animale, Libourne, 33243 France

**Keywords:** Influenza, Pig, Infection, Prevalence, Herd, Survey, Serology, Antibody

## Abstract

**Background:**

Swine influenza A viruses (swIAV) are highly contagious zoonotic pathogens that cause an acute respiratory infection in pigs, presenting substantial economic and health risks for animals and farmers. This drives the pig industry and stakeholders in animal health to monitor swIAV in livestock. In the 10 years prior to the 2009 flu pandemic, H1_av_N1 (HA-1C.2.1) and H1_hu_N2 (HA-1B.1.2.3) circulated in pig herds in France while H3N2 was not detected. The H1N1_pdm_ (HA-1A.3.3.2) lineage became enzootic after its introduction. In 2020, a new H1_av_N2 genotype (HA-1C.2.4) emerged, altering the frequencies of enzootic swIAV lineages. To support our knowledge built on event-based surveillance and gain information on swIAV infections, serological studies were conducted. Three independent surveys were performed nationally in 2008 and in 2018 (before and after the A/H1N1pdm09 pandemic, respectively), and in North West in 2022 (after the H1_av_N2 emergence in Brittany area). These surveys aimed to estimate swIAV prevalence in livestock using ELISA on fattening pigs and hemagglutination inhibition (HI) tests to determine the relative frequencies of different swIAV lineages.

**Results:**

The national seroprevalence estimated by ELISA was 91% [83–96]_CI95_ in 2008, and 87% [81–92]_CI95_ in 2018. In 2022, seroprevalence in the North West reached 91% [73–97]_CI95_. In each survey, antibodies against several swIAV subtypes were detected simultaneously by HI tests in approximately 25% of ELISA-positives batches. In 2008, anti-HA-1C.2.1 antibodies were widespread, while anti-HA-1B.1.2.3 antibodies were found in most regions except for the South West. In contrast, H3N2 seroprevalence was very low, restricted to the North East. By 2018, anti-HA-1C.2.1 antibodies remained the most prevalent and anti-H3 antibodies the weakest, but anti-HA-1B.1.2.3 prevalence had strongly decreased, while anti-HA-1A.3.3.2 antibodies were detected nationwide. In 2022, the North West showed the highest seroprevalence for H1_av_N1 (HA-1C.2.1), followed by H1_av_N2 (HA-1C.2.4), H1N1_pdm_ and finally H1_hu_N2.

**Conclusions:**

These surveys revealed a high and sustained swIAV seroprevalence, particularly in areas with a high density of pigs. They also highlighted changes in the relative frequencies of anti-HA antibodies, reflecting viral emergence and dynamics. Despite challenges in interpreting HI test results, the surveys provided valuable data, uncovering rare events (e.g. H3N2), potential undiagnosed asymptomatic cases (e.g. H1N1_pdm_), and co-circulating viruses, which may lead to genomic reassortments and the emergence of new reassortant viruses.

**Supplementary Information:**

The online version contains supplementary material available at 10.1186/s40813-025-00455-4.

## Background

Swine influenza is an acute respiratory infection, lasting less than one week and expressed clinically by dyspnea, cough, sneezing, fever and apathy [1]. It plays a key role in porcine respiratory disease complex and generates economic losses for the pig industry [2, 3]. Longitudinal field studies also revealed permanently infected herds highlighting the challenge of controlling the infection [4, 5, 6]. The highly contagious disease is caused by swine influenza A viruses (swIAV) which spread within herd by direct contact between pigs and by infectious aerosol [7]. Moreover, swIAV are zoonotic viruses and pose a pandemic risk [8, 9]. Due to their segmented genome, they can undergo reassortment when a host cell is co-infected by different viral strains. This makes pigs important “mixing vessels” for the emergence of new IAV strains [10]. In intensive pig herds, frequent contacts occur both between animals and between pigs and farmworkers, creating a favourable environment for viral persistence and zoonotic transmissions. Given the animal and public health challenges associated with swIAV, monitoring their presence in livestock is a crucial strategy to enhance our understanding of the impact of swine influenza. Globally, H1 and H3 subtypes as well as various combinations of N1 and N2 subtypes circulate worldwide in pig herds exhibiting distinct regional variations. In Europe before the 2009 flu pandemic, three subtypes from three genetic lineages circulated in pig herds: H1_av_N1 (HA-1C), H1_hu_N2 (HA-1B) and H3N2 [11, 12]. However, in France, the H3N2 virus had not been detected either by direct virological methods or by serological investigations since the late 90s [13, 14]. Following the introduction of the A/H1N1pdm09 virus into the pig population in 2010, viral diversity increased [15, 16]. From 2011 to 2018, the situation in France remained quite stable, with H1_av_N1 (HA-1C.2.1) and H1_hu_N2 (HA-1B.1.2.3) still predominating, and the presence of the H1N1_pdm_ (HA-1A.3.3.2) lineage and a few other genetic combinations detected sporadically [17, 18, 19]. In 2020, there was a marked increase in the number of clinical cases investigated, associated with the emergence of an H1_av_N2 (HA-1C.2.4) virus of a genotype newly introduced into Brittany from abroad [20]. Since then, this virus has spread throughout northwestern France, significantly changing the proportions of the swIAV strains previously circulating [21].

The virological surveillance allows an in-depth study of swIAV that are monitored both genetically and antigenically by the national reference laboratory. The swIAV are collected by a national surveillance network (RESAVIP) and other stakeholders of the pig industry. It is an event-based surveillance driven by influenza-like illness clinical signs reported by the farmer to veterinarians [18]. Asymptomatic or pauci-symptomatic cases remain undetected by this monitoring system. Despite the availability of highly sensitive commercial kits for detecting the viral genome [22], the viral excretion of an infected pig is short, which implies a short sampling timeframe for virus detection. Detecting antibodies produced by the infected host can be a way to overcome the previous limitations. There is also an interest of implementing complementary serological surveys to event-based surveillance to increase information about the burden of swIAV infections in pig populations.

Therefore, this study aimed at (i) estimating the prevalence of swIAV infections at the herd level in France and comparing its levels before and after the introduction of the pandemic A/H1N1pdm09 virus, as well as before and after the emergence of a new H1_av_N2 genotype in 2020, (ii) assessing the prevalence of the different swIAV lineages over time, both at the country level and in particular regions. Thus, three independent serological surveys were performed in 2008, 2018 and 2022, respectively, with different approaches but all targeting production farms. For each period, the rules for interpreting the results were ajusted based on the available knowledge of the reference antigens used in the hemagglunitation inhibition tests.

## Materials and methods

### Sampling plans

The target population was pigs from production herds in hexagonal France (i.e., continental France excluding Corsica) and the studied population was composed by finishing pigs (> 10 weeks old) (Table 1). At this stage, most maternally derived antibodies have disappeared, which ensures that the antibodies detected are of post-infectious origin [7]. Three blood sample collections were constituted in 2008 (survey A), 2018 (survey B) and 2022 (survey C), respectively, as follows.

### Survey A (2008)

**Fig. 1:**
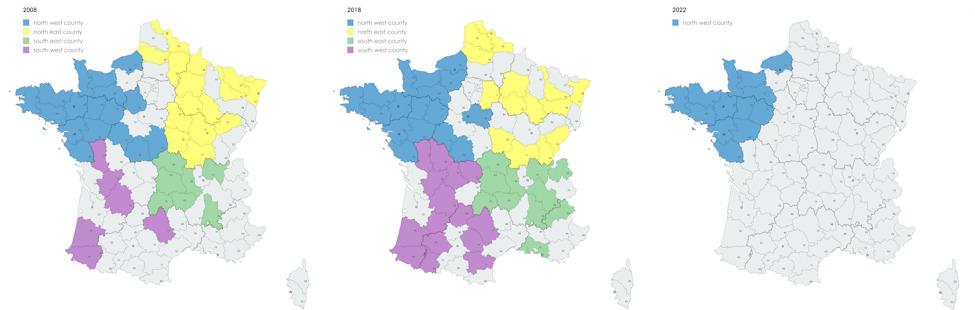
Counties where the sampled herds are located according to the three surveys and the regions. Survey A-2008 on the left; Survey B-2018 in the middle; Survey C-2022 on the right. Counties have been grouped in four geographic areas of hexagonal France (continental excluding Corsica) and coloured as follows: North West in blue; North East in yellow; South West in purple; South East in green

The sampling plan aimed to cover 95% of national production in hexagonal France by sampling pig batches at slaughterhouses using a cluster sampling design. The number of batches to be sampled at slaughterhouses was determined from the number of pigs slaughtered per year [23] and considering a minimal seroprevalence at herd level between 30% and 45% with a 20% relative precision [11, 14]. The number of pigs per batch was adapted to a minimal within-herd seroprevalence of the infection of 30% to be detected. In view of the activity of slaughterhouses in the North West, because of the high density of pig herds in this area, a pure simple random selection would have led to a very limited number of farms selected from sparsely populated areas. Hence, to get also data from those regions, we choose to select a minimum number of batches from sparsely populated regions, leading to their over-representation in the studied sample compared to their actual weight in the real population. This over representation has been corrected afterwards in the statistical analysis (see below). Thus, a total of 186 batches of 10 pigs from 24 slaughterhouses receiving batches from 45 administrative counties across the country were sampled from May 2008 to November 2009 (Table 1; Fig. 1) [23]. The sampling area was divided into four major geographical areas defined according to administrative regions: North East with Hauts-De-France, Grand-Est, Bourgogne-Franche-Comté; North West with Brittany, Normandy, Pays-de-La-Loire, Centre-Val-De-Loire; South East with Auvergne-Rhône-Alpes, Provence-Alpes-Côte d’Azur; South West with Nouvelle-Aquitaine, Occitania.

### Survey B (2018)

The samples were collected directly from farms representative of the French pig production stratified on farm type and region and selected randomly from the national database for pig herd identification (BDPORC [24]). The sample size was adapted for this survey to the estimated herd-level prevalence of the different viral subtypes based on data obtained from event-based monitoring in 2016 [18]. Therefore, sample size was calculated to allow the estimation of a prevalence between 15% and 20% for each subtype with a 20% relative precision. Thus, 487 herds were randomly selected from farrow-to-finish/post-weaning-finishing/finishing farms located in 58 administrative counties across the country in the four areas between January and June 2018 (Table 1; Fig. 1). In each herd, 10 pigs (> 10 weeks old) were sampled to detect a minimal within-herd seroprevalence of the infection of 30%.

### Survey C (2022)

As for survey A, a cluster-sampling scheme was used based on the main slaughterhouses in the area taken as clusters but the survey was restricted to the North West. The number of sampled farms was determined considering a minimal prevalence of 45% with a 20% relative precision. The number of pigs per batch was adapted to a minimal within-herd seroprevalence of the disease of 30% to be detected. Thus, 116 batches of 10 pigs from three slaughterhouses located in 13 administrative counties in Brittany and bordering regions (Normandy and Pays-de-La-Loire) were randomly sampled between February and March 2022 (Table 1; Fig. 1).

### Blood sampling

Blood samples were collected in VACUETTE^®^ 8 ml CAT Serum Separator Clot Activator tubes from pigs at slaughterhouses by technicians or on farms by veterinarians. After transportation at 4 °C, blood samples were centrifuged at the laboratory during 10 min at 4500 g and sera were stored at -20 °C until analyses.

**Table 1:** Study design of the three serological surveys conducted in pig herds in France

Year Sampling period Survey ID	Sampling process for herds	Number of sampled herds	Geographic distribution of batches	Within-herd sample size	Physiological stage
**2008** *2008-05 to 2009-11* Survey A	Cluster-based: 24 slaughterhouses	*N* = 186	45 administrative counties	10 pigs per batch	End of fattening
**2018** *2018-01 to 2018-06* Survey B	Individual-based: 487 herds	*N* = 487	58 administrative counties		Finishing pigs (> 10 weeks of age)
**2022** *2022-02 to 2022-03* Survey C	Cluster-based: 3 slaughterhouses	*N* = 116	13 administrative counties		End of fattening

### ELISA

All sera were tested by ELISA (ID Screen^®^ Influenza A Nucleoprotein Swine Indirect, Innovative Diagnostics, France) according to manufacturer’s instructions to detect antibodies directed against the swIAV nucleoprotein (NP). A serum was considered positive if its S/P ratio was > 0.4, that corresponded to an antibody titer > 1053. A batch was identified as positive if at least 1/10 serum tested positive.

### Hemagglutination inhibition tests

ELISA positive serum batches were submitted to hemagglutination inhibition (HI) tests including representative antigens of the enzootic swIAV lineages in France or Europe at the different periods of time i.e., H1_av_N1 (HA clade 1C.2.1), H1_av_N2 (HA clade 1C.2.4), H1N1_pdm_ (HA clade 1A.3.3.2), H1_hu_N2 (HA clade 1B.1.2.3) and/or H3N2 (lineage 1970.1) (Table 2) [12, 15, 17, 18, 19, 21].

Sera were treated to inactivate non-specific hemagglutination inhibitors and to remove non-specific agglutinins.

For surveys A and B, four volumes of *Vibrio cholerae* receptor-destroying enzyme were added to one volume of serum and incubated overnight at 37 °C. Then, the sera were incubated with five volumes of 1.5% sodium citrate solution for 30 min at 56 °C. Finally, chicken erythrocytes (50%) were added in 1/10 volume of the serum and incubated at 4 °C under gentle shaking for 1.5 h, before removing erythrocytes by centrifugation at 1000 g for 10 min at 4 °C. This step involved starting the serum dilution range at 1:10 [25].

For survey C, nine volumes of trypsin diluted at 1/27,000 were added to one volume of serum and incubated for 30 min at 58 °C. 225 µl of metaperiodate of sodium (0.01 M) was added to the mixture and incubated at room temperature for 15 min. After a contact of 10 min with 25 µl of glycerine (10%), a suspension of chicken erythrocytes (50%) was added in 1/20 volume of the serum and stored overnight at 4 °C before removing the erythrocytes by centrifugation. The collected supernatant corresponded to a dilution 1:20 of the serum.

HI tests were performed using four hemagglutinating units (HAU) of virus with 0.5% chicken erythrocytes according to standard procedures [26]. Two-fold treated serum dilutions were tested starting from 1:10 (surveys A and B) or 1:20 (survey C) dilution, respectively. HI titers were expressed as the reciprocal of the highest dilution of serum inhibiting four HAU. Negative and positive pig sera were included as controls. Negative sera were obtained from specific pathogen free (SPF) pigs, bred in ANSES, Ploufragan, France. Positive sera were hyper-immune sera (HIS) directed against the reference swIAVs. They were produced in SPF pigs housed in ANSES biosafety level 3 facilities, using a protocol that involved intranasal inoculation with live virus followed three weeks later by an intramuscular injection of live virus combined with an adjuvant [15, 17]. The reproducibility of the HI tests was ascertained by HIS titers obtained in homologous reactions that must be the initial expected titers +/- one dilution.

For the three surveys, a serum was declared positive towards a given reference antigen when its HI antibody titer was ≥ 20. In surveys A and B, HI titers equal to 10 were doubtful.

**Fig. 2:**
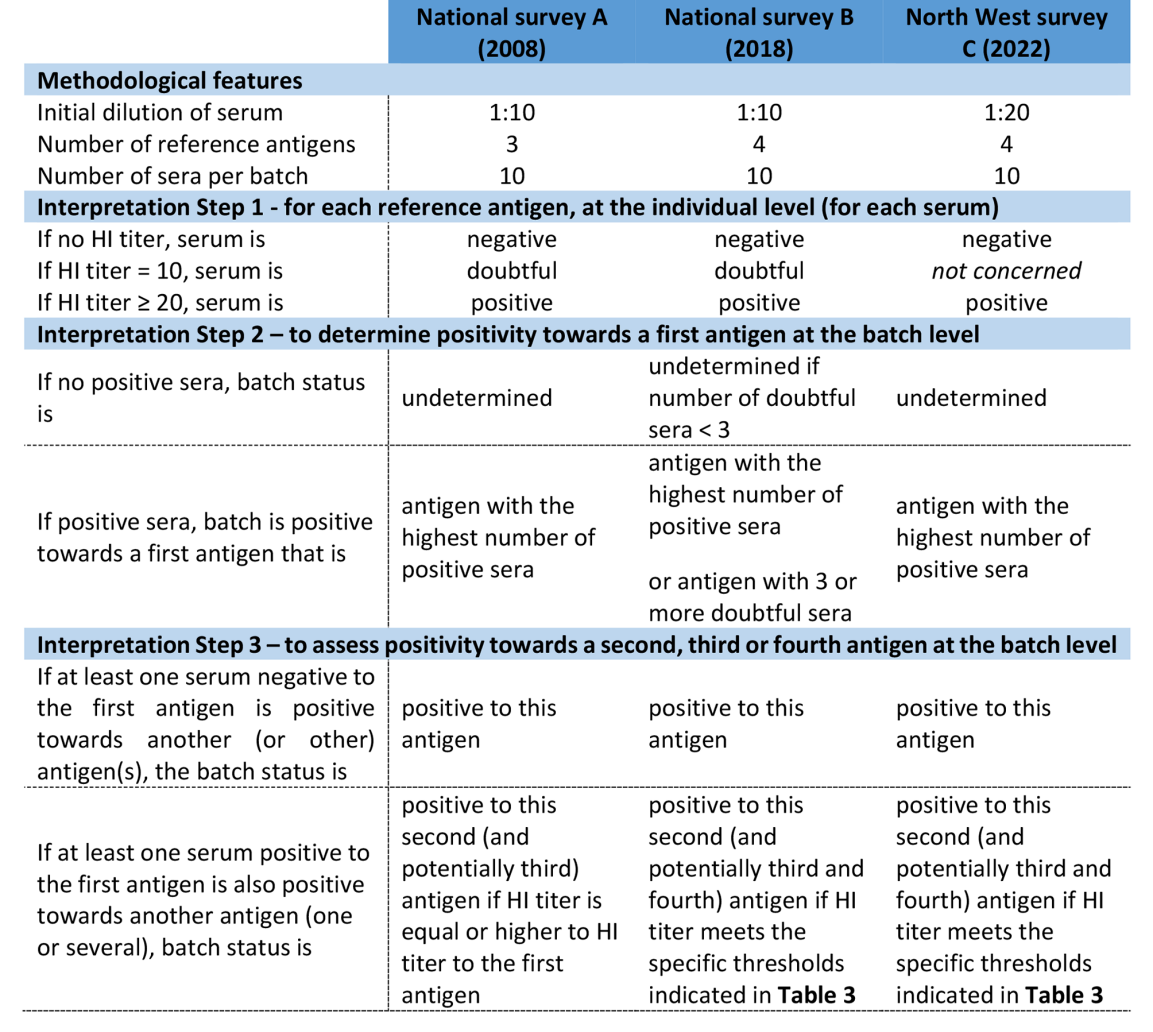
Decision-making flowchart for interpretation of HI test results from surveys **A**, **B** and **C** respectively, at the batch level

At the batch level, rules were established to interpret HI test results for each survey (Fig. 2). Previously, the European Surveillance Network for Influenza in Pigs (ESNIP) set rules to discriminate between multiple infections and cross-reactions within the HI tests [14]. These rules were used for interpretation of survey A, where three antigens were tested. However, some of these rules needed to be updated for interpretation of surveys B and C, for which four antigens were needed to be tested, in line with swIAV genetic and antigenic evolution, making the results even more complex to interpret.

For the three surveys, a batch was declared positive, in the first instance, for the antigen associated with the highest frequency of positive animals (Fig. 2).

A batch was declared as positive towards a second antigen, and a third antigen, and possibly a fourth antigen if a serum with a HI titer < 20 to the first antigen (negative serum) had an HI titer ≥ 20 (positive serum) towards another antigen. Thus, for survey A, a batch was also positive towards a second antigen if at least one serum had an equal or higher HI titer against other antigens than those against the first one (Fig. 2). For surveys B and C, the evaluation of homologous and heterologous reactions between the four tested reference antigens and HIS allowed the establishment of adjusted positivity thresholds specific to each tested lineage. Each threshold was defined based on the results of twelve independent cross-HI assays between HIS, negative serum from SPF pig and reference swine antigens [20] (supplementary table). Thus, for surveys B and C, a batch was declared as positive towards a second antigen according to the positive thresholds applied by lineage and detailed in Table 3.

Moreover, for survey B, given the number of batches exhibiting doubtful individual results (HI titers equal to 10), a batch was also considered positive towards an antigen when at least 3/10 sera were not strictly negative towards this antigen. For survey A, the doubtful results were not considered to define positive batches, in accordance with initial ESNIP rules (Fig. 2).

Finally, batches were declared of undetermined serotype if 10/10 sera obtained HI titer < 20 towards all antigens in survey A and C, and at least 8/10 sera obtained HI titer < 10 in survey B (Fig. 2).

**Table 2:** SwIAV strains used as antigens in hemagglutination Inhibition tests depending on the survey

swIAV lineage based on HA subtype and clade	2008 Survey A (National level)	2018 Survey B (National level)	2022 Survey C (North West)
H1_av_ (HA clade 1C.2.1)	A/Sw/Morbihan/0070/05	A/Sw/France/29-200272-01/2020	A/Sw/France/29-200272-01/2020
H1_av_ (HA clade 1C.2.4)	-	-	A/Sw/France/35-200154/2020
H1_pdm_ (HA clade 1A.3.3.2)	-	A/Sw/France/57-140136/2014	A/Sw/France/57-140136/2014
H1_hu_ (HA clade 1B.1.2.3)	A/Sw/Scotland/ 410440/94	A/Sw/France/35-110415/2011	A/Sw/France/35-110415/2011
H3 (1970 lineage)	A/Sw/Gent/1/84	A/Sw/France/59-150357/2015	-

**Table 3:** HI titer thresholds for positivity at the batch level in National survey B and North West survey C

	The batch is also positive against a second swIAV lineage if at least one serum has HI titer:				
**When a batch of sera is positive against a first antigen of HA lineage**:	H1_av_ (clade 1C.2.1)	H1_av_ (clade 1C.2.4)	H1_pdm_ (clade 1A.3.3.2)	H1_hu_ (clade 1B.1.2.3)	H3 (1970.1)
H1_av_ (clade 1C.2.1)	-	≥ 80	≥ 40	≥ 40	≥ 20
H1_av_ (clade C.2.4)	≥ 40	-	≥ 20	≥ 20	≥ 20
H1_pdm_ (clade 1A.3.3.2)	≥ 40	≥ 40	-	≥ 40	≥ 20
H1_hu_ (clade 1B.1.2.3)	≥ 20	≥ 40	≥ 40	-	≥ 40
H3 (1970.1)	≥ 20	≥ 80	≥ 20	≥ 20	-

The thresholds were determined according to known antigen-antibody cross-reactions (supplementary table)

### Calculation of seroprevalence values and statistical analyses

The design of the surveys (cluster sampling for surveys A and C, unequal weighting of observations among sampled counties when compared to the actual population) was taken into account to obtain population-based estimates of the seroprevalence values. An adjustment of the seroprevalence results was applied for each county based on the real number of pig farms with fattening pigs per county in the study area, using the official database (BDPORC 2012, 2018 or 2022 for surveys A, B and C, respectively). As the database for the French pig production in 2008 was no longer available at the time of the analyses, the 2012 database was used to survey A. As for survey B farm characteristics were available because of individual-based sampling from the national database, hence the real distribution of the different farm types (farrow-to-finish, wean-to-finish or finishing farm) was also used as a parameter of adjustment in the estimations. The analyses were carried out with the R Survey package version 4.4 [27]. The regional seroprevalence values were established accounting for the herd location in one of the four areas previously defined, i.e., North East, North West, South East or South West (Fig. 1).

Moreover, for national survey B conducted in 2018, a logistic regression was applied to the adjusted sample (function svyglm using the R Survey package) to calculate the odds of a herd being seropositive given several variables (herd type, region and age of sampled pigs).

For comparisons of quantitative data of ELISA titers, Wilcoxon Rank-Sum test was applied and significant differences were defined if p-value was < 0.05 (R version 4.1.2).

## Results

### High swIAV seroprevalence levels were sustained over time

**Fig. 3:**
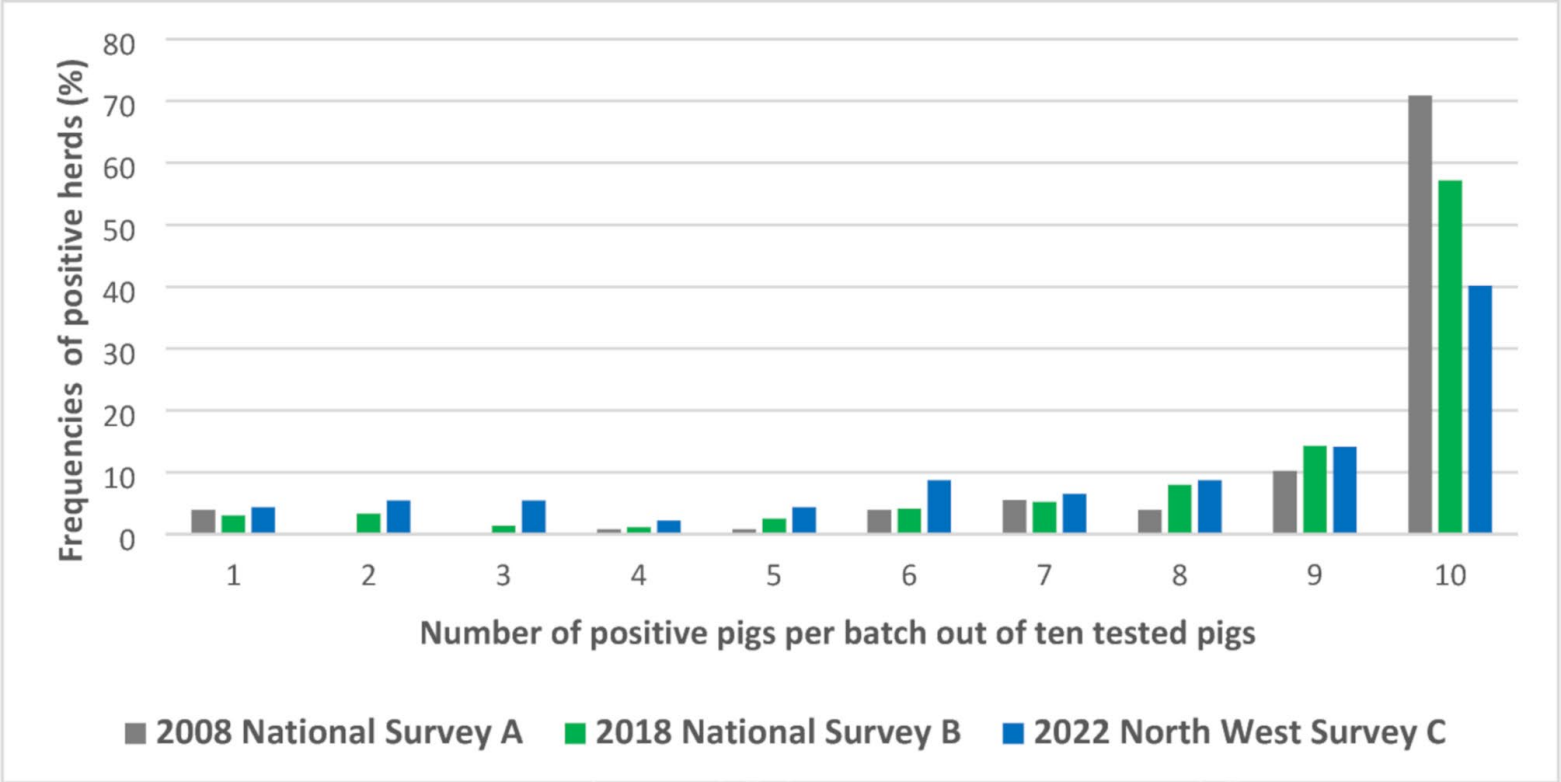
Within-herd distribution of positive samples. The number of positive pig(s) per batch (among 10 tested) is given in relation to the frequencies of ELISA-positive herds in national surveys conducted in 2008 (survey **A**) and 2018 (survey **B**), as well as in the survey conducted in the North West in 2022 (survey **C**)

The national seroprevalence value was estimated to be 91% in 2008 (survey A) and 87% in 2018 (survey B) (Table 4). The within-herd seropositivity distributions ranged from 10 to 100% and were not considered statistically different in both national surveys (χ2 p-value = 0.13, Fig. 3). A within-herd seropositivity two-times higher than the expected one was observed, with at least 6 out of 10 pigs testing positive in 94% of herds in 2008, 89% in 2018 and 78% in 2022 (Fig. 3).

At the regional level, the highest seroprevalence value was detected in the North West in both 2008 and 2018 periods (94% and 88%, respectively), at similar rates than that found in this region in 2022 (91%) (Table 4). In the North East, an important evolution occurred between 2008 and 2018, rising from 52 to 82%. A similar increase, from 28% in 2008 to 68% in 2018, was measured in the South East, whereas the seroprevalence in the South West remained stable between 2008 and 2018, around 49% of positive herds.

**Table 4:** National and/or regional estimated seroprevalence values of swIAV infections in France in 2008, 2018 and 2022

Study area	2008 National Survey A *N* = 186	2018 National Survey B *N* = 487	2022 North West Survey C *N* = 116
Hexagonal France	**91%** [83–96] _CI95%_ n_pos_=127	**87%** [81–92] _CI95%_ n_pos_= 364	-
North East	**52%** [18–84] _CI95%_ n_pos_=8	**82%** [63–93] _CI95%_ n_pos_=33	-
North West	**94%** [90–97] _CI95%_ n_pos_=105	**88%** [82–92] _CI95%_ n_pos_=295	**91%** [73–97] _CI95%_ n_pos_=92
South East	**28%** [6–70] _CI95%_ n_pos_=5	**68%** [39–87] _CI95%_ n_pos_=14	-
South West	**49%** [31–68] _CI95%_ n_pos_=9	**49%** [42–56] _CI95%_ n_pos_=22	-

N = number of tested herds. Confidence interval (CI) at 95% is given into brackets; n_pos_=number of positive herds by ELISA

### Estimated seroprevalence values of the different swIAV lineages changed over time

**Fig. 4:**
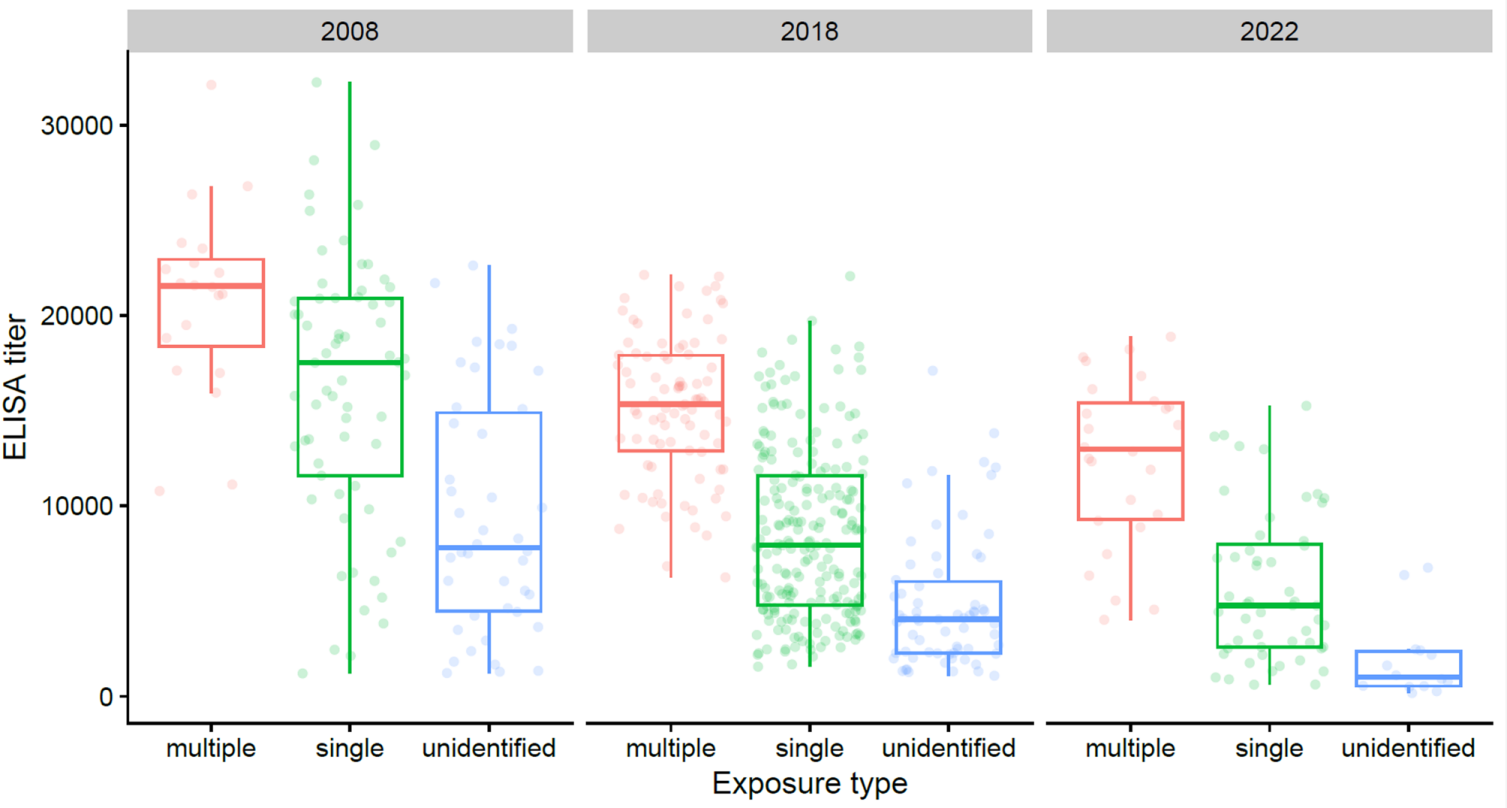
Distribution of ELISA titers according to the type of swIAV lineage exposure in 2008 (national survey **A**), 2018 (national survey **B**) and 2022 (North West survey **C**). Each plot represents the batch average ELISA titer and is colored in red for the multiple swIAV lineage exposure, in green for the single swIAV lineage exposure and in blue for the unidentified swIAV lineage exposure

The frequencies of batches found infected by only one virus subtype were estimated below 50% in both national surveys (Table 5). Anti-HA antibodies against two or three different antigens were detected in around a quarter of cases (Table 5). The presence of anti-HA antibodies directed towards only one antigen was shown in half of the farms tested in the North West area in 2022 while a third showed several swIAV lineage exposures. The mean ELISA titers themselves could not be compared between surveys as they used different batches of the commercial ELISA kit. However, it can be noticed that, in all surveys, the mean ELISA titers of positive herds were higher when HI tests revealed multiple lineage exposures (Table 5; Fig. 4). Thus, statistical differences in average ELISA titers were observed between groups of herds exposed to multiple swIAV lineages, a single lineage or an unidentified lineage with the same trend seen across all periods (p-value < 0.05). Herds with unidentified swIAV exposure had significantly lower average ELISA titers compared to those exposed to one or more known lineages (p-value < 0.05).

**Table 5:** Estimated frequencies of multiple, single or unidentified swIAV lineage exposure in 2008 (national survey A), 2018 (national survey B) and 2022 (North West survey C)

swIAV lineage exposure	2008 National Survey A	2018 National Survey B	2022 North West Survey C
**Multiple swIAV lineage exposure**	**21%** [16–29]_CI95_ n_pos_=20 **20,862**	**26%** [22–29]_CI95_ n_pos_=87 **15,168**	**30%** [23–37]_CI95_ n_pos_=26 **12,403**
**Single swIAV lineage exposure**	**47%** [37–58]_CI95_ n_pos_=65 **16,210**	**49%** [45–53]_CI95_ n_pos_=211 **8516**	**50%** [40–59]_CI95_ n_pos_=52 **5675**
**Unidentified swIAV lineage exposure**	**23%** [16–32]_CI95_ n_pos_=42 **9519**	**13%** [12–14]_CI95_ n_pos_=66 **4836**	**11%** [7–18]_CI95_ n_pos_=14 **1902**

Confidence interval (CI) at 95% is given into brackets; n_pos_=number of positive batches by ELISA; Mean ELISA titers of positive batches are given in bold in the third line

**Fig. 5:**
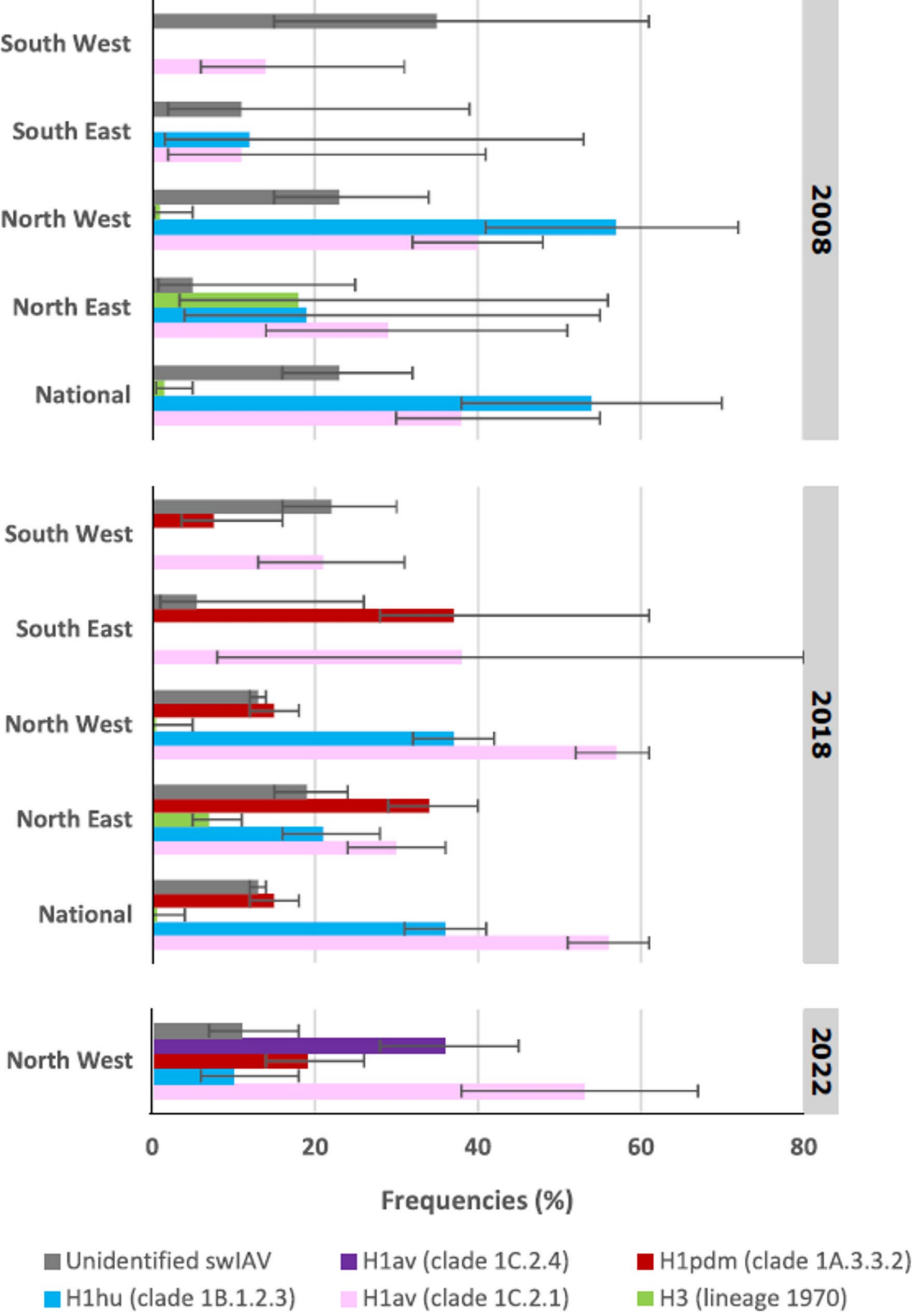
Estimated seroprevalence values of the swIAV lineages at the national and regional levels in 2008, 2018, and in North West in 2022. The four areas were North East, North West, South East and South West in 2008 (up), 2018 (middle) and only North West in 2022 (down). The swIAV lineages identified were H1_av_ clade 1C.2.1 in pink, H1_hu_ clade 1B.1.2.3 in blue, H1_pdm_ clade 1A.3.3.2 in red, H3 in green and H1_av_ clade 1C.2.4 in purple. The frequency of unidentified swIAV lineage was in grey. The confidence interval at 95% was identified by the black bar

At the national level, the most prevalent swIAV lineage infecting pigs in production herds in 2008 was the H1_hu_N2 (HA-clade 1B.1.2.3), followed by the H1_av_N1 lineage (HA-clade 1C.2.1) (Fig. 5). In 2018, trends have reversed, with a higher proportion of batches seropositive towards H1_av_N1 than H1_hu_N2. In 2018, antibodies towards a third lineage, i.e., H1_pdm_ clade 1A.3.3.2, counted for 15% of positive batches. The seroprevalence of H3N2 was very low, but not negative, in both national surveys.

Estimations of regional seroprevalence revealed markedly contrasting situations depending on the area. In the North East area, all three swIAV lineages tested in 2008 were detected, with the highest seroprevalence observed for H1_av_N1, followed by H1_hu_N2 and H3N2 lineages, which exhibited similar seroprevalence levels (Fig. 5). In the North West area, the H1_hu_N2 lineage showed the highest seroprevalence, followed by H1_av_N1, whereas the prevalence of the H3N2 lineage was estimated to be very low. In the South East, antibodies were detected against only two swIAV lineages, H1_av_N1 and H1_hu_N2, with similar prevalence values. In the South West, only antibodies against H1_av_N1 lineage were detected.

In 2018, the situation evolved as infections due to the H1N1_pdm_ virus were evidenced in the four areas of the territory. The North East area remained a region where all tested swIAV lineages were detected, with the highest seroprevalence values towards H1N1_pdm_, closely followed by H1_av_N1, then H1_hu_N2 and more weakly H3N2 (Fig. 5). In the North West area, the seroprevalences of the H1_av_N1 and H1_hu_N2 lineages prevailed over those of H1N1_pdm_ and H3N2. In the South East and South West areas, the pigs were only exposed to H1_av_N1 and H1N1_pdm_. However, their seroprevalence levels were similar in South East but different in South West.

The survey conducted in the North West area in 2022 showed that a high seroprevalence value towards H1_av_N1 (HA-clade 1C.2.1) was maintained over time, followed by the newly introduced H1_av_N2 (HA-clade 1C.2.4), and H1N1_pdm_ also, while the seroprevalence towards H1_hu_N2 strongly diminished compared to data from previous survey in 2018 (Fig. 5).

The proportion of cases where the swIAV subtype responsible for the presence of antibodies in sera remained undetermined, counted for less than a quarter of positive cases in surveys conducted at the national level (Table 5). However, the frequency of unidentified subtype varied at the regional level depending on the survey (Fig. 5). In 2008, it was very high in South West but very low in North East. In 2018, the highest frequency of unidentified swIAV subtype was observed again in South West but the lowest was found in South East (Fig. 5). By contrast, it markedly decreased in North West and increased in North East. In North West, the frequency of unidentified positive cases measured in 2022 remained similar to that calculated in 2018.

### Impact of geographic area, pig production type and age of pig on swIAV seroprevalence in 2018

In 2018, herds in South West were evaluated to be 5 times (OR = 0.21 [0.08–0.55]_CI95_) less affected by swIAV infection than those in North East, which was considered as the reference in the statistical model (Table 6). The risk of influenza infection in North West and North East, as well as in South East and North East, were not statistically different (p-value > 0.05).

Herds located in North West were 3 times more exposed to the risk of H1_av_N1 virus infection (OR = 3.11 [2.24–4.31]_CI95_) and twice more exposed to the risk of H1_hu_N2 virus infection (OR = 2.18 [1.49–3.20]_CI95_) than those from North East (Table 6). However, they were 3 times less impacted by H1N1_pdm_ infection (OR = 0.33 [0.24–0.46]_CI95_). Also, pigs seemed to be less impacted by H1N1_pdm_ infection in South West than in North East (OR = 0.16 [0.07–0.34]_CI95_).

At the national level, the post-weaning-finishing type of pig production was estimated 3 times less infected than farrow-to-finish type (OR = 0.33 [0.23 − 0.46] _CI95_) (Table 6). This seemed to be especially the case in North West area (OR = 0.32 [0.20 − 0.51] _CI95_), but in North East and South East, the finishing production type was more infected compared to the farrow-to-finish herds (OR = 6.80 [1.72 − 26.88]_CI95_ and 4.35 [2.31 − 8.19]_CI95_respectively).

Finally, two ranges of age ([10;15] and [≥ 16] weeks, respectively) were compared and it appeared that the oldest pigs had twice more risk than the youngest ones to be seropositive (OR = 2.43 [1.07 − 5.55]_CI95_) (Table 6).

**Table 6:** Impact of geographic area, pig production type and age of sampled pigs on swIAV seroprevalence in 2018

Epidemiological factors	OR	2.5% OR	97.5% OR	*p*-value
*Geographic area on swIAV positivity*				
North East	**1**	-	-	-
North West	**1**.**60**	0.56	4.55	NS
South East	**0**.**45**	0.11	1.89	NS
South West	**0**.**21**	0.08	0.55	0.01
*Geographic area on H1*_*av*_ *N1 positivity*				
North East	**1**	-	-	-
North West	**3**.**11**	2.24	4.31	< 0.0001
South East	**1**.**44**	0.26	8.02	NS
South West	**0**.**62**	0.36	1.07	NS
*Geographic area on H1*_*hu*_ *N2 positivity*				
North East	**1**	-	-	-
North West	**2**.**18**	1.49	3.20	< 0.0001
South East	**0**	0	0	< 0.0001
South West	**0**	0	0	< 0.0001
*Geographic area on H1N1*_*pdm*_ *positivity*				
North East	**1**	-	-	-
North West	**0**.**33**	0.24	0.46	< 0.0001
South East	**1**.**12**	0.46	2.75	NS
South West	**0**.**16**	0.07	0.34	< 0.0001
*Production type on swIAV positivity*				
Farrow-to-finish (*n* = 200)	**1**	-	-	-
Finishing (*n* = 177)	**0**.**63**	0.38	1.04	NS
Post-weaning-finishing (*n* = 110)	**0**.**33**	0.23	0.46	< 0.0001
*Age of pigs on swIAV positivity*				
]10 ;15] WOA (*n* = 46)	**1**	-	-	-
≥ 16 WOA (*n* = 224)	**2**.**43**	1.07	5.55	0.035

Odd Ratio (OR), 95% confidence interval and p-value were calculated by logistic regression using data from national survey B conducted in 2018. NS: not significant, WOA: week of age, n: number of cases

## Discussion

National serological surveys conducted in 2008 and 2018 revealed that swIAV infections were largely affecting the pig population in hexagonal France at both periods. The swIAV seroprevalence in 2008, which reached 91%, was comparable to that reported in Spain during the same time (93.9%), based on a cross-sectional survey conducted on 98 farms across ten regions of the country [28]. In 2018, a comparably high national seroprevalence of 87% was reported in France. In both national surveys, the highest swIAV seroprevalence was observed in the North West, the country’s main pig-producing region [18]. By 2022, the seroprevalence had returned to a similarly high level. Upward trends were observed between 2008 and 2018 in North East and South East, which could reflect much more swIAV infection cases due to the introduction of H1N1pdm in 2009–2010 although the data must be interpreted with caution given the large confidence intervals of 2008 survey in these two regions. The high within-herd frequency of seropositive pigs observed in the three surveys was not surprising since swine influenza is a very contagious disease, as supported by the high reproduction number estimated in field condition [4] and as previously calculated in experimental conditions [7]. The target population was pigs over 10 weeks of age but not sows nor piglets, to discard any interference of serological responses linked to breeding herd vaccination or maternally derived antibodies. In national survey B-2018, we observed a higher risk of antibody detection in oldest pigs (≥ 16 weeks). Logically, there is an increasing probability of swIAV exposure linked to the age, as the oldest animals could have been infected with different swIAV subtypes successively. This is in line with a study conducted in 2019–2023 in commercial farrow-to-finish pig farms in Greece that estimated variable seroprevalence according to the age group of pigs [29]. We also observed that herd type had an impact at the national level, with a higher risk of antibody detection in farrow-to-finish herds compared to post-weaning-finishing herds. This increasing risk may be due to self-sustaining infections, which are influenced by production systems and intensive herd management, and are more likely reported in herds with breeding animals [4, 7, 18].

By analyzing ELISA-positive batches in HI tests, we were able to highlight changes in anti-HA antibody relative frequencies over time, reflecting viral emergence and dynamics. As passive surveillance of swIAV infections was not established nationwide in 2008 and data collection was restricted to the North West [12], national survey A enhanced knowledge of circulating virus subtypes in France and revealed notable regional specificities. Thus, anti-HA-1C.2.1 antibodies were widespread, while anti-HA-1B.1.2.3 antibodies were found in most regions except for the South West. Moreover, a low H3N2 seroprevalence was evidenced while restricted to the North East, in line with data from Belgium, a boarder country where the H3N2 virus was prevalent at this time [14]. While overall seroprevalence remained unchanged in 2018 as compared to 2008, the frequencies of the various viral lineages changed significantly, notably due to the introduction of the H1N1pdm virus from 2009-2010. Thus, by 2018, whereas anti-HA-1C.2.1 antibodies remained the most prevalent and anti-H3 antibodies the weakest, anti-HA-1B.1.2.3 prevalence had strongly decreased, while anti-HA-1A.3.3.2 antibodies were detected nationwide. These results confirmed the H1N1pdm virus has disseminated in the pig population in France, as observed worldwide, especially in areas previously free of swIAV as in Norway [30, 31]. Dissemination of H1N1pdm could at least partially explain the apparent increase in swIAV seroprevalence in North East and South East of France between 2008 and 2018, where it might have competed with the H1_hu_N2 virus. Globally, the relative frequencies of the different anti-HA antibodies detected nationwide in 2018 qualitatively mirrored the distribution of virus lineages identified through the event-based surveillance from 2010 onward, including sporadic cases of H3N2 infections in the North East [15, 16, 17, 18, 19, 21, 32]. Nevertheless, H3N2 and H1N1pdm infections were found in higher levels in serology compared to viral detection, especially in the North West, demonstrating the value of serological investigations in revealing rare events (H3N2 infections) and/or infections that may be pauci- or asymptomatic (H1N1pdm infections) and which are not well captured by clinical monitoring. In 2022, the seroprevalences of viral lineages in the North West of France have evolved as compared to 2018, in line with the emergence of the new H1_av_N2 (HA-1C.2.4) lineage in Brittany in 2020 [20, 21], whose prevalence has been estimated slightly lower than that of the H1_av_N1 virus. By contrast, the event-based surveillance carried out in 2021 showed a higher proportion of influenza cases due to H1_av_N2 (1C.2.4) than H1_av_N1 [21]. The H1_av_N2 (1C.2.4) virus, which is genetically and antigenically distant from other H1_av_-1C.2 viruses detected in France, was responsible for an epizootic, probably because it was able to escape pre-existing population immunity [21, 33]. Interestingly, although this emerging virus was responsible for many clinical cases, serological data from survey C revealed that the oldest H1_av_N1 continued circulating in the pig population, potentially with less marked symptoms than the new genotype, leading to fewer diagnostic investigations from practitioners. Again, these results confirmed that passive surveillance allowed describing swIAV strains in circulation and their relative frequencies in clinical cases, but it was not sufficient to determine their respective impacts. As highlighted by others, active monitoring in enzootic herds, including detection of early infections in farrowing sector by testing the dams and their suckling pigs using alternative samples such as oral fluids could be proposed as complementary surveillance strategies [34]. 

To simplify interpretations, detection of anti-HA antibodies in sera was associated to previous infection with a swIAV belonging to the same virus lineage than the corresponding reference antigen. For instance, reactivity towards the H1_av_N1 (HA-1C.2.1) reference antigen was considered having followed infection by a virus strain of this lineage, whereas it might also result from infection by a H1_av_N2 virus strain bearing a H1 gene from the HA-1C.2.1 clade. Thus, infections with reassortant viruses could not be distinguished using HI tests, but they probably accounted for a very small proportion of cases if any, since data from virological surveillance identified H1_hu_N1 (HA-1B.1.2.3), H1_av_N2 (HA-1C.2.1), H1_av_N1 (HA-1C.2.2) or H1_pdm_N2 (HA-1A.3.3.2) virus strains only sporadically during the studied periods [12, 17, 21].

As reported in previous studies, we evidenced here the presence of anti-HA antibodies directed against different swIAV subtypes simultaneously [11, 14]. Exposure to several lineages was found in around a quarter of farms nationwide, and in slightly higher proportions in the North West of France. These results emphasized the consecutive or simultaneous circulation of different swIAV lineages in herds. These situations are conducive to co-infections and may lead to the generation of new viruses through genetic reassortment. In fact, new reassortant viruses that may constitute a threat for animal and public health were sporadically picked up by event-based surveillance, especially in the North West [17, 21, 35]. Since the levels of post-infectious immune responses may depend on the time and order of successive infections due to antigenic sin and prime-boost reactions [4, 36, 37], biases in the respective prevalences of the different lineages tested might exist, but they were impossible to assess here. In any case, these serological investigations showed the magnitude of co-circulations, confirming a non-negligible risk of emergence of reassortants.

The analytical approach was consistent across all surveys, with ELISA as a primary method for detecting anti-NP antibodies, providing insights into disease prevalence, followed by HI tests, which were more challenging to interpret but permitted identification of main swIAV lineages responsible for the infections. However, despite the new information and additional knowledge acquired from this study gathering three surveys, we must point out a few limitations to serological investigations as conducted here.

The three sampling operations were independent, conducted years apart by different teams. Therefore, study designs, sample sizes and geographical areas were different, thus we could not compare their results strictly as numerical data of lineage’s prevalence.

Other difficulties in interpreting and comparing data from HI tests came from methodological differences between the surveys and the absence of standardized rules in consequence. The first difference concerned the method used to treat sera for non-specific agglutinins before contact with reference antigens. In view of the results obtained in the three surveys respectively, we would recommend using a treatment that allows HI test starting with a serum dilution range from 1:10 to enhance sensitivity. Thus, numbers of sera with HI titers equal to 10 could be taken into account to declare a batch positive towards an antigen if necessary, as done in survey B.

Indeed, a weakness in our study could be the proportions of unidentified cases among ELISA-positive batches. The lack of identification of anti-HA antibodies may be related to a weaker sensitivity of HI tests compared to ELISA, since most unidentified cases in HI tests corresponded to batches exhibiting the lowest average ELISA titers. However, it remains possible that the lack of reaction in HI tests was linked to the tested antigens. Selecting the appropriate antigens is a critical step in conducting HI tests, as it ensures accurate detection of antibodies with a good sensitivity. Achieving very good antigenic matching between reference strains and antibodies generated by field infections remains a significant challenge. In each survey, the antigens were selected based on knowledge of circulating viruses at the European and/or French levels [11, 12, 15, 16, 17, 18, 19, 21]. However, as HI tests are time-consuming work we could not test more than four antigens head-on for each survey within the framework of this study. That is why we did not introduce the H3N2 subtype in survey C-2022 because of its very low expected prevalence, opting to focus on the new H1N2 suspected to constitute the major change that occurred in the North West area between 2018 and 2022. Thus, we cannot exclude part of serological response towards the H3N2 subtype among the low proportion of unidentified lineage exposure cases in survey C. However, as far as possible, the same antigen was used for a given lineage throughout the different surveys. For example, the H1_av_N1 reference strain from 2020 was used in both surveys B-2018 and C-2022 because of a low antigenic drift within 1C.2.1 strains during this period. To accurately interpret HI titers, it is essential to assess both the antigenic distances and the cross-reactivity between the reference antigens. For survey A-2008, we followed ESNIP guidelines [14], but adjustments were made in surveys B-2018 and C-2022 due to the inclusion of additional reference antigens. As a result, positivity thresholds for HI titers were not uniformly applied across the three surveys, but were instead adapted based on cross-reactivity levels within each reference panel to ensure consistent interpretation.

Curiously, the highest frequencies of unidentified lineage exposure were obtained in the South West region in both national surveys A-2008 and B-2018. Additional HI test using an H1_av_N2 (HA-1C.2.4) antigen, which was punctually isolated locally in 2015 [38] was performed on sera from survey B-2018; however, this test did not result in the detection of corresponding anti-HA-1C.2.4 antibodies (data not shown). Another sub-sample of 18 unidentified batches from survey B-2018, issued from the four regions, were tested against a contemporary human H3N2 antigen, but none of them led to a readable HI titer as well (data not shown). It could be informative to test other IAV, e.g. a contemporary human H1N1 or strains of avian origin, which might have crossed the species barrier. However, the list of possibilities is extensive, that is why these undetermined cases recall the importance to set up virological surveillance as comprehensive as possible to help in defining the most appropriate panels of antigens to be included in HI tests. To improve sensitivity in identifying swIAV antibodies detected in pig sera, micro-neutralization assays could be another option, but they also require a suitable panel of live viral strains.

## Conclusions

While serological surveys alone cannot fully identify all swIAV strains circulating in pig herds or track viral evolution, they provide valuable complementary data to virus detection and characterization. This information is crucial for estimating infection prevalence at national or regional levels, detecting asymptomatic cases, and identifying rare events. Despite the challenges in conducting these surveys and interpreting HI tests, the findings reported here highlight the importance of regularly performing serological surveys to enhance understanding of swIAV subtype distribution and support event-based surveillance, which remains essential.

## Electronic supplementary material

Supplementary table

## Data Availability

The datasets used and/or analysed during the current study are available from the corresponding author on reasonable request.
