# Supplementary material for: Trends in seroprevalence of influenza A virus infections in pigs in France (2008–2022)
Source: Porcine Health Manag. 2025 Jul 28;11:42. doi: 10.1186/s40813-025-00455-4 (PMC12306050; doi:10.1186/s40813-025-00455-4)
Supplement: Supplementary file 1 — Supplementary table [file 40813_2025_455_MOESM1_ESM.docx]

**Supplementary table:** Cross-hemagglutination inhibition (HI) assays using hyper-immune sera (HIS) and homologous reference antigens.

| **Antigens** |  | **HI titers of**  **swine hyper-immune sera containing antibodies directed against** | | | | | | **SPF pig serum** | | |  |
| --- | --- | --- | --- | --- | --- | --- | --- | --- | --- | --- | --- |
|  | **Subtype** | H1_av_N1 | H1_av_N2 | H1N1_pdm_ | H1_hu_N2 | H3N2 | | | - | |  |
|  | **Clade** | 1C.2.1 | 1C.2.4 | 1A.3.3.2 | 1B.1 | 1970.1 | | | - | |  |
| A/sw/France/29-200272/2020 (H1_av_N1) | | **[640-1280] 693** | [10-20] 14 | [10-20] 16 | [<10-10] 5 | | [<10-<10] <10 | | | [<10-<10] <10 | |
| A/sw/France/35-200154/2020 (H1_av_N2) | | [10-40] 22 | **[1280-2560] 1600** | [10-20] 17 | [10-20] 13 | | [10-40] 17 | | | [<10-<10] <10 | |
| A/sw/France/57-140136/2014 (H1N1_pdm_) | | [10-20] 18 | [<10-<10] <10 | **[640-1280] 907** | [10-20] 17 | | [<10-<10] <10 | | | [<10-<10] <10 | |
| A/Sw/France/35-110415/2011 (H1_hu_N2) | | [10-20] 18 | [<10-<10] <10 | [10-20] 11 | **[640-2560] 1440** | | [<10-<10] <10 | | | [<10-<10] <10 | |
| A/Sw/France/59-150357/2015 (H3N2) | | [<10-<10] <10 | [<10-<10] <10 | [<10-10] 3 | [<10-20] 7 | | **[2560-5120] 4480** | | | [<10-<10] <10 | |

HIS were produced in specific pathogen free (SPF) pigs inoculated with representative swIAV strains. The table reports the range of HI titers and the mean HI titers obtained from 12 independent assays. The titers obtained in homologous reactions are in bold. Sera from non-inoculated SPF pigs were included as negative controls.
